# Speciation Characterization and Environmental Stability of Arsenic in Arsenic-Containing Copper Slag Tailing

**DOI:** 10.3390/molecules29071502

**Published:** 2024-03-27

**Authors:** Mu You, Yunhu Hu, Chuncai Zhou, Guijian Liu

**Affiliations:** 1 School of Biology Engineering, Huainan Normal University, Huainan 232001, China; youmu@ustc.edu.cn; 2 School of Chemistry and Materials Engineering, Huainan Normal University, Huainan 232001, China; huyunhu@ustc.edu.cn; 3 School of Resources and Environmental Engineering, Hefei University of Technology, No. 193, Road Tunxi, Hefei 230009, China; 4 School of Earth and Space Sciences, University of Science and Technology of China, Hefei 230026, China; lgj@ustc.edu.cn

**Keywords:** mineralogy, chemical speciation, environmental mobility, arsenic, copper slag tailing

## Abstract

The increasing presence of arsenic-containing impurities within Cu ores can adversely affect the smelting process and aggravate the environmental impact of slag tailing. This study investigates the geochemical, mineralogical, and chemical speciation characteristics to better understand the association and environmental stability of metal(loid)s in copper slag tailing. The results indicate that the predominant chemical compositions of the selected slag tailing are Fe_2_O_3_ (54.8%) and SiO_2_ (28.1%). These tailings exhibit potential for multi-elemental contamination due to elevated concentrations of environmentally sensitive elements. Mineral phases identified within the slag tailings include silicate (fayalite), oxides (magnetite and hematite), and sulfides (galena, sphalerite, arsenopyrite, and chalcopyrite). The consistent presence of silicate, iron, arsenic, and oxygen in the elemental distribution suggests the existence of arsenic within silicate minerals in the form of Si-Fe-As-O phases. Additionally, arsenic shows association with sulfide minerals and oxides. The percentages of arsenite (As(III)) and arsenate (As(V)) within the selected slag tailings are 59.4% and 40.6%, respectively. While the slag tailings are deemed non-hazardous due to the minimal amounts of toxic elements in leachates, proper disposal measures should be taken due to the elevated carbonate-bound levels of As and Cu present in these tailings.

## 1. Introduction

Copper slag tailings, primarily stemming from pyrometallurgical processes, predominantly consist of a silicate matrix [1]. It is estimated that 2–2.2 tons of slag tailing are generated for every ton of copper produced, and approximately 40 million tons of slag are generated from global copper production [2,3,4]. Unfortunately, slag tailings are often directly deposited near industrial hubs without proper disposal practices [5]. Recent research has explored reusing slag tailings as additives for construction materials, concretes, and abrasives, demonstrating excellent soundness characteristics, abrasion resistance, and stability in thermally treated by-products [6,7,8,9,10,11]. However, to ensure environmental safety and inertness, thorough assessments are essential for potential recycling and engineering applications of slag tailings, especially concerning the elevated concentrations of metal(loid)s such as As, Pb, Cu, and Zn within these tailings [12,13].

The exposure of landfill-disposed and engineered applications of slags to various bio-hydro-climatic conditions has been noted in research [14,15]. Long-term progressive biogeochemical weathering and physical erosion of these materials can induce chemical and structural alterations, potentially mobilizing metallic elements and ultimately contributing to the deterioration of environmental quality [16]. Many studies have investigated the mobility mechanisms of metallic elements during weathering, highlighting that the redistribution of these elements depends on several parameters. These parameters include the association and chemical speciation of metallic elements, the stability (lattice energy) of their host minerals, and the prevailing leaching environment (pH; redox potential) [17,18,19]. Simultaneously, the interactions between metallic elements and bulk compositions, particularly iron (Fe), play a significant role in effectively immobilizing the metallic elements at the slag–water interface [20]. A consensus has emerged that dissolved metallic elements can be trapped and immobilized through the precipitation of secondary phases [21]. Consequently, conducting detailed chemical, mineralogical, and textual characterizations of slag tailings becomes imperative to elucidate the principal factors influencing the mobility of metallic elements during disposal and engineering applications.

Arsenic, a naturally occurring toxic element, can be present in copper ores in various forms. While tennantite (Cu_12_As_4_S_13_) and enargite (Cu_3_AsS_4_) are known arsenic-bearing minerals associated with certain copper ores [22,23], their occurrence is not universal across all copper ore types. It is important to recognize that arsenic can also be found in other minerals that are more commonly associated with copper ores or even in minerals not primarily known for containing arsenic, such as pyrite (FeS_2_). Pyrite, despite not being a primary source of arsenic, can still host elevated levels of this element, contributing to the overall arsenic content in copper ore environments. Within copper ores, arsenic undergoes redistribution, primarily among flue dust and slag, with approximate ratios of 76–85% and 7–17%, respectively [4]. Consequently, the captured flue dust, containing elevated arsenic concentrations, is recycled back into the smelting process for the high content of Cu (up to 40%). This recycling process ensures the maintenance of the pyrometallurgical balance and prevents the degradation of Cu matte quality [24,25]. The overall concentration of arsenic in slag tailings can reach up to 7.59%, posing a potential environmental pressure [4]. The transformation behavior and biotoxicity of arsenic differ among the various phases in which arsenic occurs within slag tailings [26,27]. The presence of arsenic in sulfide minerals exhibits relatively high susceptibility to alteration, potentially increasing its mobilization, whereas arsenic bound in silicate minerals may be retained within the silicate lattice [28]. Therefore, research focusing on the association and chemical speciation of metallic elements, particularly arsenic, within slag tailings holds significant importance.

The main objectives of this study are to (1) determine the bulk chemical compositions and the mineralogical and micromorphological characterizations of the selected arsenic-containing slag tailing; (2) elucidate the association and chemical speciation of arsenic within the selected slag tailing; (3) evaluate the potential eco-environmental risks caused by metallic elements in slag tailing. The findings obtained from these objectives hold significance in providing crucial insights for both the proper disposal and utilization of slag tailings.

## 2. Results and Discussion

### 2.1. Physico-Chemical Property of Copper Slag Tailing

Understanding the chemical, mineralogical, and textural properties of slag tailings is crucial in evaluating potential biological toxicity and the environmental stability of sensitive elements (such as As, Pb, and Zn), pivotal for appropriate slag disposal and utilization [29,30]. Variation in smelted ores, fluxes, additives, and pyrometallurgical conditions (furnace, atmosphere, temperature, and cooling time) causes the bulk chemical, mineralogical, and textural characterizations to vary among different smelting plants, ultimately affecting the environmental stability of the slag tailing [31]. The bulk chemical compositions of the selected slag tailing, presented in Table 1, primarily comprise Fe_2_O_3_ (54.8%) and SiO_2_ (28.1%), with minor quantities of Al, Ca Na, and K oxides, which is different from previous studies in Australia [21], the USA [29], Spain [31], Portugal [32], and Poland [15] (Table 1). The elevated Fe content in the slag tailing is attributed to the presence of bornite (FeCu_5_S_4_) and chalcopyrite (CuFeS_2_) in the Cu concentrate [33]. Silica content in the slag tailing originates from both the primary ore and the added silica flux during the smelting process [33]. The presence of alkali oxides in the slag tailing is associated with the concentrations of these compounds in gangue minerals of the Cu concentrate [34]. Additionally, the incorporation of Ca-based oxides is aimed at reducing silica viscosity and enhancing Cu separation efficiency during smelting processes [4].

Table 1 presents the range or average values of mineral elements (zinc, arsenic, copper, lead, chromium, and nickel) from different regions or sources. There are significant variations in the mineral element content among different regions or sources, which may be influenced by factors such as geology, ore deposit type, mining, and smelting processes. In this study, the minor elements are listed in descending order as follows: Zn > As > Cu > Pb > Cr > Ni. The notably elevated levels of these elements within the slag tailing raise concerns about the potential environmental impacts during both disposal and utilization stages. Comparative analysis with other copper slags from various sources [15,21,29,31] reveals that the selected slag tailing exhibits particularly high as content, warranting significant attention due to its environmental implications. However, it is noteworthy that the lowest concentration of Cu detected in the selected slag tailing signifies the exceptional efficiency of Cu metallurgical processes employed in the designated furnace.

**Figure 1:**
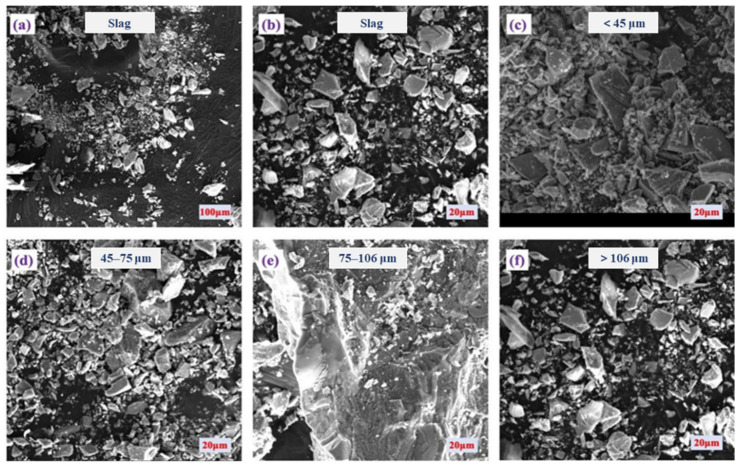
The morphology characterization of the slag tailing at different scales: (**a**) overview; (**b**) feature tailings; (**c**) size < 45 μm; (**d**) size in the range of 45–75 μm; (**e**) size in the range of 75–106 μm; (**f**) size > 106 μm.

The selected slag tailing underwent screening using sieves of 150 (106 μm), 200 (75 μm), and 325 (45 μm) mesh sizes to assess particle size distribution. The fractions of particle sizes within the selected slag tailing were categorized as follows: <45 μm (25.4%), 45–75 μm (52.4%), 75–106 μm (14.3%), and >106 μm (7.9%), respectively. The selected slag tailing predominantly exists as fine particles (<75 μm), constituting up to 77.8%, with the primary presence observed in the 45–75 μm range (52.4%). Figure 1 presents a series of scanning electron microscopy (SEM) images that depict the slag tailing particles segregated by size fractions, demonstrating the variations in morphology and texture that are pivotal to comprehending the slag’s physical characteristics and its potential environmental interactions. Figure 1a offers a broad overview of the heterogeneous nature of the slag tailing sample. Figure 1b zooms in to reveal particles with angular and irregular shapes. In Figure 1c, particles less than 45 μm are shown, characterized by a predominantly amorphous texture. Figure 1d captures the 45–75 μm fraction, where particles exhibit sharper edges and a reduced amorphous quality. Figure 1e represents the 75–106 μm fraction, highlighting a textural and formative transition. Lastly, Figure 1f focuses on particles exceeding 106 μm, showcasing larger, more distinct crystalline structures. Collectively, the slag tailing has angular grains across various size fractions, attributed to the milling process. Moreover, finer amorphous materials are notably increased with decreasing particle sizes. These images elucidate the dependency of particle shape and texture on particle size, factors that are crucial in influencing the leachability of metallic and metalloid constituents from the slag.

**Figure 2:**
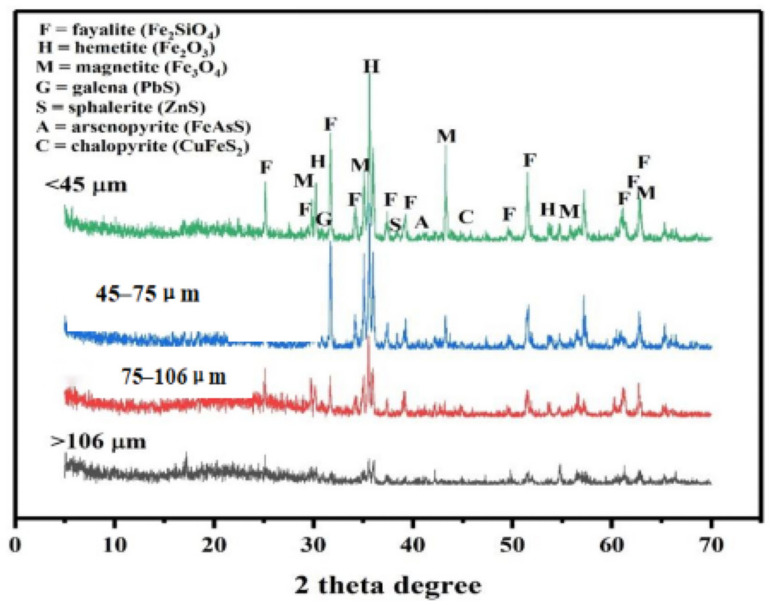
The mineralogical characterizations of the selected slag.

Metallic elements (Cu, Pb, As, and Zn) are primarily associated with copper sulfides within the Cu concentrate [35,36]. These elements are released and redistributed during high-temperature smelting processes as their host minerals undergo complex physico-chemical transformations—volatilization, decomposition, oxidation, reduction, and crystallization—resulting in the formation of Cu matte and new phases in the smelting slag [10,37]. The mineral phases within the slag, considered synthetic analogs of natural minerals, are formed through anthropogenic activities, dictated by smelting conditions (temperature and cooling speed) and the slag’s chemical compositions [38]. Slower-cooled slags exhibit more diverse phase varieties compared to their faster-cooled counterparts, attributed to closer equilibrium in phase crystallization [3,39]. The equilibrium of phase crystallization at slow cooling rates leads to a more diverse phase composition in slag due to prolonged exposure to smelting conditions. Meanwhile, smelting temperature significantly influences the phase compositions of slag, resulting in the formation of various minerals like spinel, melilite, olivine, pyroxene, and glass, each formed at distinct temperatures and within different durations [2]. The mineral phases present in the slag tailing encompass a range of categories, including silicates (olivine and pyroxene), oxides (magnetite and hematite), sulfides (chalcocite, chalcopyrite, galena, sphalerite, and bornite), pure metals, intermetallic compounds, and glass matrixes [1]. The mineralogical characteristics, depicted in Figure 2 for different size fractions, primarily feature fayalite, hematite, magnetite, galena, sphalerite, arsenopyrite, and chalcopyrite. Among these, fayalite, hematite, and magnetite predominantly represent Fe-based minerals, while fayalite and silicate glasses form the primary silicate phases. Additionally, sulfides (galena, sphalerite, arsenopyrite, and chalcopyrite) are consistently observed in minor quantities within the selected slag tailing, aligning with findings from other furnace studies [15,32]. Despite sulfides being volumetrically minor phases in slag tailing, these minerals should be taken fully into consideration for potential environmental risks [40]. Upon comparing the mineralogical distribution among different size fractions, the peaks of fayalite and hematite show an increasing trend with decreasing size fractions, indicating a preference for these secondary phases to exist in finer particles.

### 2.2. Association and Chemical Speciation of Arsenic in Copper Slag Tailing

In the smelting furnace, arsenic, as an impurity, undergoes removal via volatilization and slagging processes [41]. During high-temperature smelting, arsenic sulfide and elemental arsenic are oxidized, transforming into arsenic trioxide [25]. This trioxide then undergoes volatilization and condensation onto fine particulate surfaces during smoke dust collection. In an oxidizing environment, arsenic trioxide may further react with oxygen to form non-volatile arsenic pentoxide [14]. It has been reported that the volatilization ratio is approximately 76–85%, with 7–17% of arsenic existing in slag during flash smelting processes [4]. For the high Cu content (up to 40%) in the collected dust, the collected arsenic-containing dust is recycled in the smelting furnace and results in the deterioration of Cu matte. Consequently, the smelting slag serves as the primary discharge outlet for various pyrometallurgical impurities (As, Zn, Pb, and Ni), impacting the pyrometallurgical quality. The release and environmental mobility of elements from slags are controlled by various factors, i.e., the geochemical/mineralogical compositions of slags, the association and speciation of elements, and the disposal processes [22,23]. Among them, understanding the association and chemical speciation is crucial, offering significant insights into the toxicity and bioavailability of arsenic. To unravel these aspects, SEM-EDS, XPS, and sequential chemical extraction procedures are applied.

**Figure 3:**
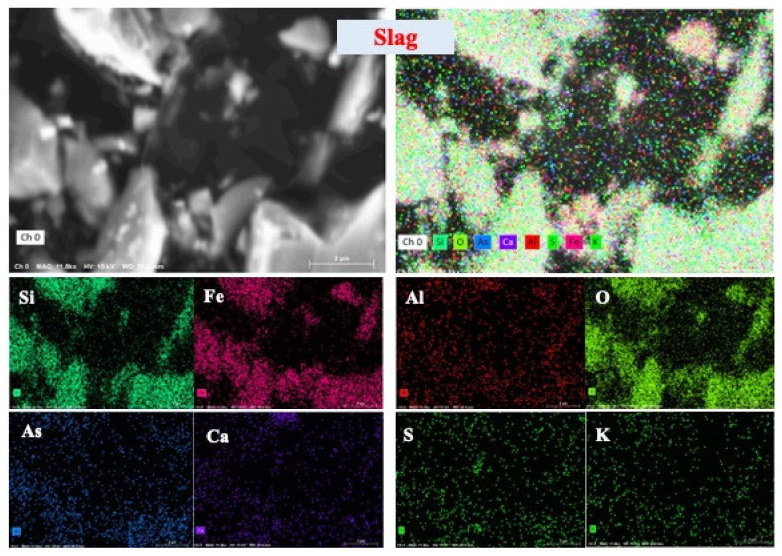
Two-dimensional distribution of elements in slag tailing.

The two-dimensional distribution images in Figure 3 and Figure S1 showcase the elemental distribution in the slag tailing, particularly highlighting arsenic’s predominance in finer particles (<75 μm), as evident from Figure S1. The intersection of silicate, iron, arsenic, and oxygen in the EDS images suggests a potential association of arsenic with silicate minerals, forming Si-Fe-As-O phases [29]. According to the distributions of elements in different size fractions (Figure S1), the Si-Fe-As-O phases are mostly found in the fine particulates (<75 μm). Many studies reported that As-O phases disseminated in the silicate minerals are the primary Si-Fe-As-O phases in slag tailing [25,28]. Meanwhile, observations of overlapping areas between arsenic and sulfur in the selected slag tailing indicate the presence of arsenic sulfides. Consequently, arsenic primarily exists within silicate minerals, sulfide minerals, and oxides within the slag tailing, manifesting its diverse associations within these mineralogical components.

**Figure 4:**
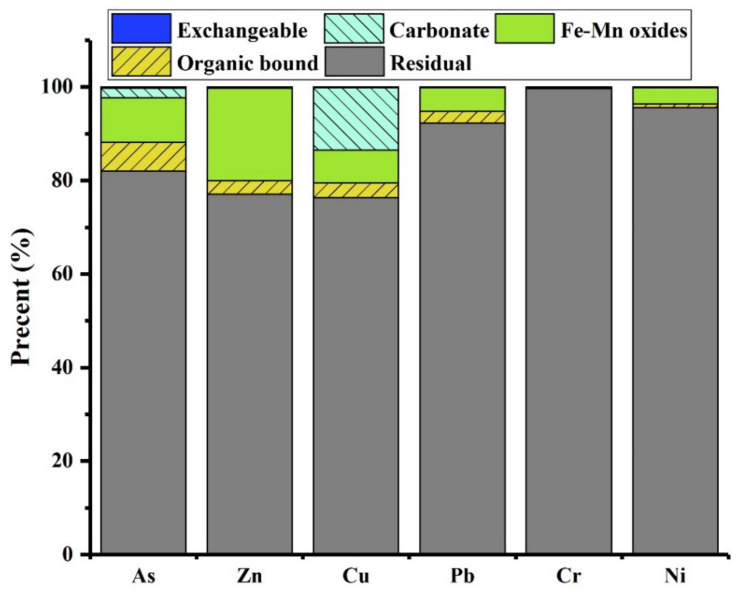
Association of toxic elements in selected slag.

The associations of arsenic, as determined by sequential chemical extraction (Figure 4), reveal its primary presence in residual form (82.1%), Fe-Mn oxide-bound fractions (9.6%), and organic matter-bound states (6.2%). The elevated fractions of As in residue and Fe-Mn oxides suggest that As is mainly associated with silicate minerals. Additionally, the presence of arsenic associated with organic matter may be attributed to the adsorption onto unburned organic materials.

**Figure 5:**
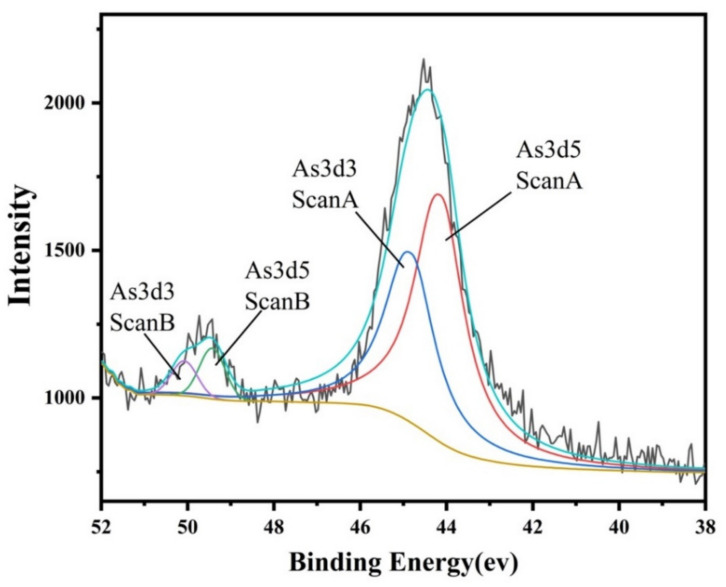
Chemical speciation and valence state of arsenic in selected slag.

Understanding the chemical speciation of arsenic is pivotal for assessing its toxicity and bioavailability in slag tailing. According to the valence states of As, compounds can be clustered into arsine (As^−3^), elemental As (0), arsenite (As^+3^), and arsenate (As^+5^) [29]. Among them, arsenite (As (III)) and arsenate (As (V)) are the main valence states of As in slag tailing [42]. The different colors likely represent the fitted peaks for different electronic states of arsenic was shown in Figure 5. The distinct peaks at 49.98 eV and 44.53 eV correspond to metallic arsenic in the As 3d peaks. It is clearly seen that As in slag tailing could exist in both As(III) and As(V) with the proportions of 59.4% and 40.6%, respectively. For the high toxicity of As(III), the environmental risks of slag tailing caused by As deserve further investigation.

### 2.3. Potential Environmental Risks of Copper Slag Tailing

Under natural weathering conditions, the environmental risks associated with environmentally sensitive elements in slag tailing are contingent upon both total concentration and chemical speciation [14]. Assessing the total concentrations of elements in slag tailing is crucial for understanding pollution levels. However, the environmental stability and toxicity of elements are predominantly determined by their leaching potential and duration.

The results of CN-SWEP and TCLP leaching tests (Table 2) indicate that the leaching of elements (As, Cu, Zn, Pb, Ni, and Cr) in the selected slag tailing remains significantly below legislative limits, classifying it as non-hazardous inert waste. Additionally, the sequential chemical extraction procedure results serve as an indicator for evaluating potential environmental risks. The Risk Assessment Code (RAC) assigns safety to elements when the exchangeable and carbonate-bound fractions constitute less than 1% of the total value. Conversely, elements pose a strong environmental impact (very high risk) when these fractions exceed 50% of the total value. RAC values falling within 1–10%, 11–30%, and 31–50% are categorized as low, medium, and high risk, respectively. The fractionation characteristics of elements in slag tailing are presented in Figure 4. The selected environmentally sensitive elements primarily exist in residual form. The RAC values of Zn, Pb, Cr, and Ni reveal that the environmental impacts of these elements could be regarded as negligible. However, speciation patterns of As and Cu show a low risk and medium risk, respectively. Therefore, the environmental impacts induced by these elements in slag tailing should be taken into consideration during disposal and utilization.

The discrepancy between the leaching test results and the RAC model could be attributed to the interaction between exchangeable elements and Fe. The presence of high levels of Fe, mainly as Fe^2+^, facilitates its rapid oxidation to Fe^3+^ in the water phase, causing other elements to co-precipitate with Fe during the formation of iron (hydr)oxide colloids. This process effectively reduces the dissolved elements (Cu and As) during leaching tests.

## 3. Materials and Methods

### 3.1. Sampling

**Figure 6:**
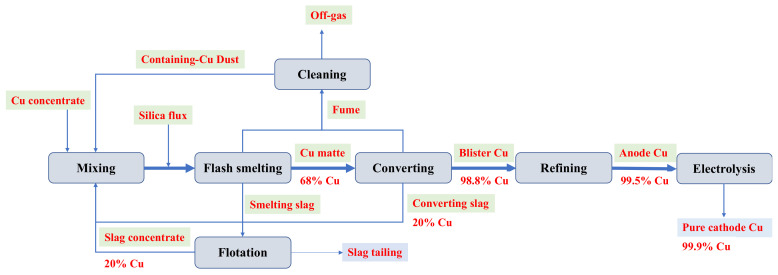
Flowsheet of pyrometallurgical copper production.

The slag tailing was collected from a Cu smelter in China, where the Cu concentrate comprised covellite (CuS), bornite (FeCu_5_S_4_), chalcopyrite (CuFeS_2_), and Cu-As bearing minerals including tennantite (Cu_12_As_4_S_13_) [43]. Concentration, smelting, converting, and refining/electrolysis are critical steps in pyrometallurgical copper production, the process of which is presented in Figure 6.

The Cu concentrate, mixed with silica flux, undergoes smelting in an Outokumpu flash smelting furnace at a temperature of approximately 1450–1550 °C, producing an intermediate product (Cu matte with 68% Cu content). The addition of silica flux could improve the separation of Fe during smelting. Simultaneously, Ca-based oxides are used to lower silica viscosity and improve Cu separation efficiency. The Cu matte is subsequently converted into blister Cu (98.8% Cu) in a flash converting furnace. Waste slags from the smelting and converting furnaces undergo granulation and reprocessing through milling and flotation. This process divides the waste slags into slag concentrate (recycled in the smelting furnace due to its high Cu content of 20%) and slag tailing. The slag tailing, dewatered, is stockpiled near the industry site. The flue gas emitted from the furnaces is filtered through pollution control devices, capturing flue dust with high Cu content (40%) but also containing substantial impurities, notably As, which can potentially disrupt the smelting process and exacerbate slag generation. The emissions from the process adhere to set standards before being discharged. Blister Cu is then refined and processed via electrolysis to yield pure cathode Cu with a purity of 99.9%. Notably, in the pyrometallurgical copper production flow, slag tailing stands as the sole discharge outlet for various pyrometallurgical impurities. In this study, approximately 5 kg of fresh slag tailing was collected from the stockpile and promptly sealed in a plastic bag to prevent contamination. The selected slag tailing was air-dried and screened using sieves of 150 (106 μm), 200 (75 μm), and 325 (45 μm) mesh for subsequent analysis.

### 3.2. Mineralogical and Micromorphological Analysis

The selected slag tailing was measured by using X-ray powder diffraction (XRD, RIGAKU, D/Max-2500, Bruker Corporation, Madison, WI, USA) within a 2θ range of 5–70°, employing a step increment of 0.01°, equipped with a Cu-Kα radiation source. Identification of minerals in each sample was accomplished by referencing the ICDD Powder Diffraction File. For detailed micromorphological analysis and elemental distribution within the slag tailing, a Tescan MIRA3 field emission scanning electron microscope (SEM, Brno, Czech Republic) equipped with four energy dispersive X-ray spectroscopy (EDX) detectors was employed. The SEM operated at an accelerating voltage of 30 kV and a beam current of 10–10 nA. Additionally, a full-color cathodoluminescence detector with a wavelength range spanning from 200 nm to 850 nm was utilized. The oxides of major elements within the selected slag tailing were determined by X-ray fluorescence spectrometry (XRF, PANalytical Axios mAX, PANalytical B.V., Almelo, The Netherlands). The detection limits of the XRF instrument for the elements of interest range from a few parts per million (ppm) to several tens of ppm, allowing for the precise quantification of trace elements in our slag tailing samples. The concentration of arsenic was measured by using inductively coupled plasma emission spectroscopy (model NWR 213-7900 ICP-MS), with limits as low as 0.01 μg/L for elements such as arsenic. The samples were thoroughly mixed to eliminate any heterogeneity, and 0.45 μm membrane filtration was employed to remove any particulate matter that could potentially interfere with the analysis or damage the instrument.

### 3.3. Chemical Analysis

The oxides of major elements within the selected slag tailing were determined by using XRF. To extract the toxic elements (As, Cu, Cr, Ni, Pb, and Zn), a microwave digestion procedure was executed utilizing an acidic mixture solution of HCl:HNO_3_:HF at a 3:3:2 ratio. After digestion and quantification, the concentrations of the selected toxic elements were determined by using ICP-MS. To ensure the accuracy of both the extraction and analytical procedures, NIST standard reference materials and blank samples were employed. The precision attained for all of the selected elements falls within ±5 wt. %.

### 3.4. Speciation Analysis

The determination of element association and speciation behavior employed both direct and indirect approaches. Both X-ray photoelectron spectroscopy (XPS) and the sequential chemical extraction method were utilized to ascertain the chemical forms and speciation characteristics of As and other elements present in the selected slag tailing. XPS patterns were recorded by a Thermo-VG Scientific (Waltham, MA, USA) ESCALAB 250 spectrometer with Al Kα X-rays as the sputtering source at a power of 150 W. The standard calibration for the binding energy utilized 284.6 eV for C (1 s). High-resolution scans of As 3d were performed with an energy step size of 0.05 eV.

The classic sequential chemical extraction method, as outlined by Tessier [44], was employed to determine the speciation behavior of As and other elements in the selected slag tailing. This method categorized the speciation of elements into five fractions, as detailed in Table 3. The concentration of toxic elements within each fraction was subsequently determined by using ICP-MS.

### 3.5. Environmental Stability Analysis

In order to evaluate the potential environmental impacts of toxic elements in the slag tailing, the solid waste extraction procedure for leaching toxicity (CN-SWEP) according to Chinese standard [29] was conducted to determine the potential leaching toxicity of toxic elements. The samples were leached at an L/S (liquid/solid) ratio of 10 L/kg in an acidic mixture solution (pH = 3.20 ±0.05) at 23 ± 2 °C for 18 ± 2 h. The acidic solution was prepared by blending H_2_SO_4_ and HNO_3_ at a ratio of 2:1. The concentrations of toxic elements in leachates were analyzed by using ICP-MS.

Additionally, the toxicity characteristic leaching procedure (TCLP), recommended by the Environmental Protection Agency (EPA), was utilized to assess the environmental risks associated with toxic elements in the selected slag tailing. This procedure involved the extraction of samples using a CH_3_COOH solution (pH = 4.95 ± 0.05) with an L/S ratio of 20 L/kg. The samples were continuously agitated at 100 ± 10 rpm for 18 ± 2 h. Solution pH was adjusted using HCl and HNO_3_. The leachate obtained through centrifugation at 4500 rpm for 10 min was subsequently analyzed by using ICP-MS.

## 4. Conclusions

The bulk chemical compositions of the selected slag tailing are Fe_2_O_3_ (54.8%) and SiO_2_ (28.1%), with minor quantities of Al, Ca Na, and K oxides. In comparison with other Cu smelting furnaces, the low Cu content in the slag tailing indicates the excellent pyrometallurgical efficiency of the selected smelting furnace. The mineral phases in slag tailing are fayalite, hematite, magnetite, galena, sphalerite, arsenopyrite, and chalcopyrite. The peaks of fayalite and hematite increase with the decreasing size of the fractions, suggesting that these second phases are preferable to exist in fine particles. As-O phases disseminated in the silicate minerals are the primary Si-Fe-As-O phases in the slag tailing. Meanwhile, arsenic is also associated with sulfide minerals and oxides. As in the slag tailing could exist in both As(III) and As(V) with the proportions of 59.4% and 40.6%, respectively. The results of the CN-SWEP and TCLP leaching tests suggest that the selected slag tailing could be considered non-hazardous inert waste. However, the high proportions of carbonate-bound phases of As and Cu indicate that the release of As and Cu from the slag tailing may lead to potential environmental issues if no countermeasures are adopted.

The potential for arsenic and copper release, particularly from carbonate-bound phases, underscores the need for effective management strategies to mitigate environmental risks. It is crucial to extend our investigations to include the evaluation of other toxic metals within slag tailings. Such studies will enhance our understanding of the environmental impacts of slag tailings and inform the development of more comprehensive management and remediation strategies. This direction not only aligns with our findings but also opens avenues for future research aimed at minimizing the ecological footprint of mining activities.

## Figures and Tables

**Table 1:** The chemical compositions of the selected copper slag.

Major Elements (wt. %)	SiO_2_	Al_2_O_3_	Fe_2_O_3_	CaO	Na_2_O	K_2_O
This study	28.1 ± 0.54	3.80 ± 0.22	54.8 ± 2.54	3.58 ± 0.68	0.70 ± 0.49	1.35 ± 0.10
Australia [21] (Lottermoser, 2005)	28.7–35.8	3.12–5.41	31.5–43.5	9.12–15.5	0.04–0.26	0.05–1.16
The USA [29]	14.5–20.3	1–4.9	21–37	0.83–4.1	0.1–1.9	0.23–1.2
Spain [31]	13.8–68.7	0.01–15	6.72–50.6	0.2–7.85	0.11–1.25	0.01–3.05
Portugal [32]	28.5–34.4	1.51–1.97	58.2–58.5	4.95–6.28	0.02–0.15	0.22–0.26
Poland [15]	31.9–70.7	3.84–11.9	5.58–51.1	0.59–1.68	0.15–2.05	1.25–4.37
Miner Elements (mg/kg)	Zn	As	Cu	Pb	Cr	Ni
This study	2034 ± 65	901 ± 124	563 ± 187	341 ± 25	72.7 ± 21.8	35.8 ± 17.7
Australia [21]	12,266–58,560	24–635	1410–8586	90–51,620		0–25
The USA [29]	2300–19,700	0.5–2	1900–13,500	8.1–47	40–276	2.8–27
Spain [31]	58–1423	58–8623	1400–280,600	16–4562	118–653	53–217
Portugal [32]	>10,000	180	3280	>5000		
Poland [15]	1294–9360	3–315	3030–13,400	11–738		

**Table 2:** Leaching results of toxic elements from selected copper slag obtained by CN-SNEP and TCLP (mg/L).

Methods	As	Cu	Zn	Pb	Ni	Cr
CN-SNEP	2.512	24.150	2.478	0.274	0.112	0.034
TCLP	2.529	8.056	0.521	0.013	0.020	ND
China limitation	5	100	100	5	5	1
USA limitation	5	20	250	5	5	1

ND: the concentration of the substance was below the detection limit of analytical method.

**Table 3:** Sequential extraction procedure.

Step	Extraction Procedure	Speciation
1	Samples of 1.0 g were extracted with 10 mL 1.0 M MgCl_2_ (pH = 7.0) under room temperature for 1 h; suspension was achieved by centrifugation at 3500 rpm for 20 min.	Exchangeable
2	The residual solid from step 1 was extracted with 10 mL 1 M sodium acetate (pH = 5.0) under room temperature and agitated continuously for 5 h; suspension was achieved by centrifugation at 3500 rpm for 20 min.	Carbonate-bound
3	The residual solid from step 2 was treated with 20 mL 0.04 M NH_2_OH·HCl in 25% (*v*/*v*) under room temperature and agitated continuously for 6 h; suspension was achieved by centrifugation at 3500 rpm for 20 min.	Fe-Mn oxide-bound
4	The residual solid from step 3 was treated with 3 mL 0.02 M HNO_3_ and 5 mL 30% H_2_O_2_ (pH = 2.0) under 85 °C for 2 h. A second 3 mL aliquot of 30% H_2_O_2_ (pH = 2.0 with HNO_3_) was then added under 85 °C and agitated for 3 h. After cooling, 5 mL of 3.2 M NH_4_OAc in 20% (*v*/*v*) HNO_3_ was added and the sample was diluted to 100 mL and agitated continuously for 30 min.	Organic matter-bound
5	The residual solid from step 4 was digested with a HCl-HNO_3_-HF mixture according to the procedure used for bulk samples	Residual

## Data Availability

The datasets used and/or analyzed during the current study are available from the corresponding author upon reasonable request.

## Supplementary Materials

The following supporting information can be downloaded at: https://www.mdpi.com/article/10.3390/molecules29071502/s1, Figure S1: Two-dimensional distribution of elements in different size fractions of slag tailing.
