# Supplementary material for: Speciation Characterization and Environmental Stability of Arsenic in Arsenic-Containing Copper Slag Tailing
Source: Molecules. 2024 Mar 27;29(7):1502. doi: 10.3390/molecules29071502 (PMC11012958; doi:10.3390/molecules29071502)
Supplement: Supplementary file 1 [file molecules-29-01502-s001.zip › molecules-2913946-supplementary.pdf]

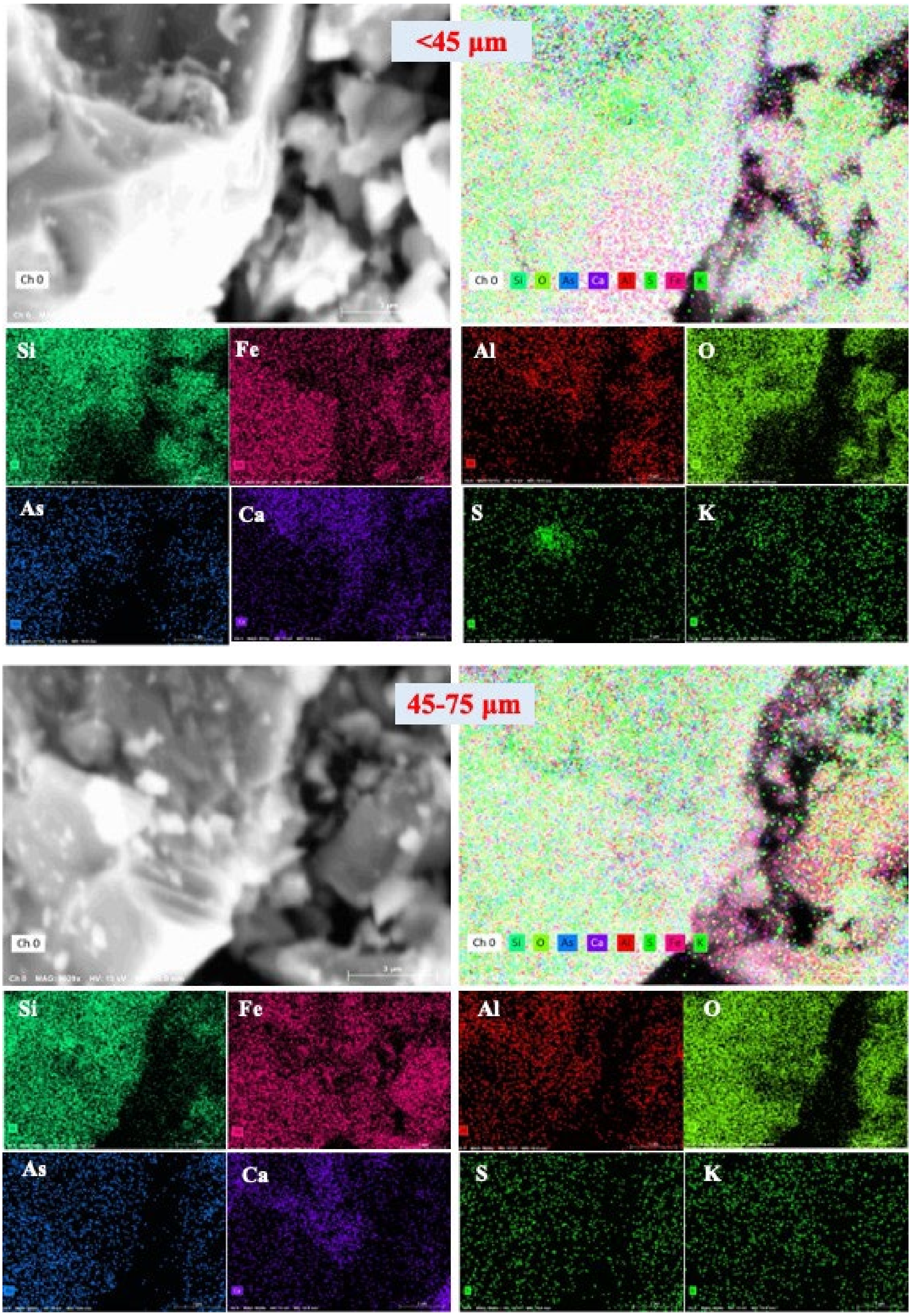
<45 μm
Ch 0
Si
O
As
Ca
Al
S
Fe
K
45-75 μm
3 μm

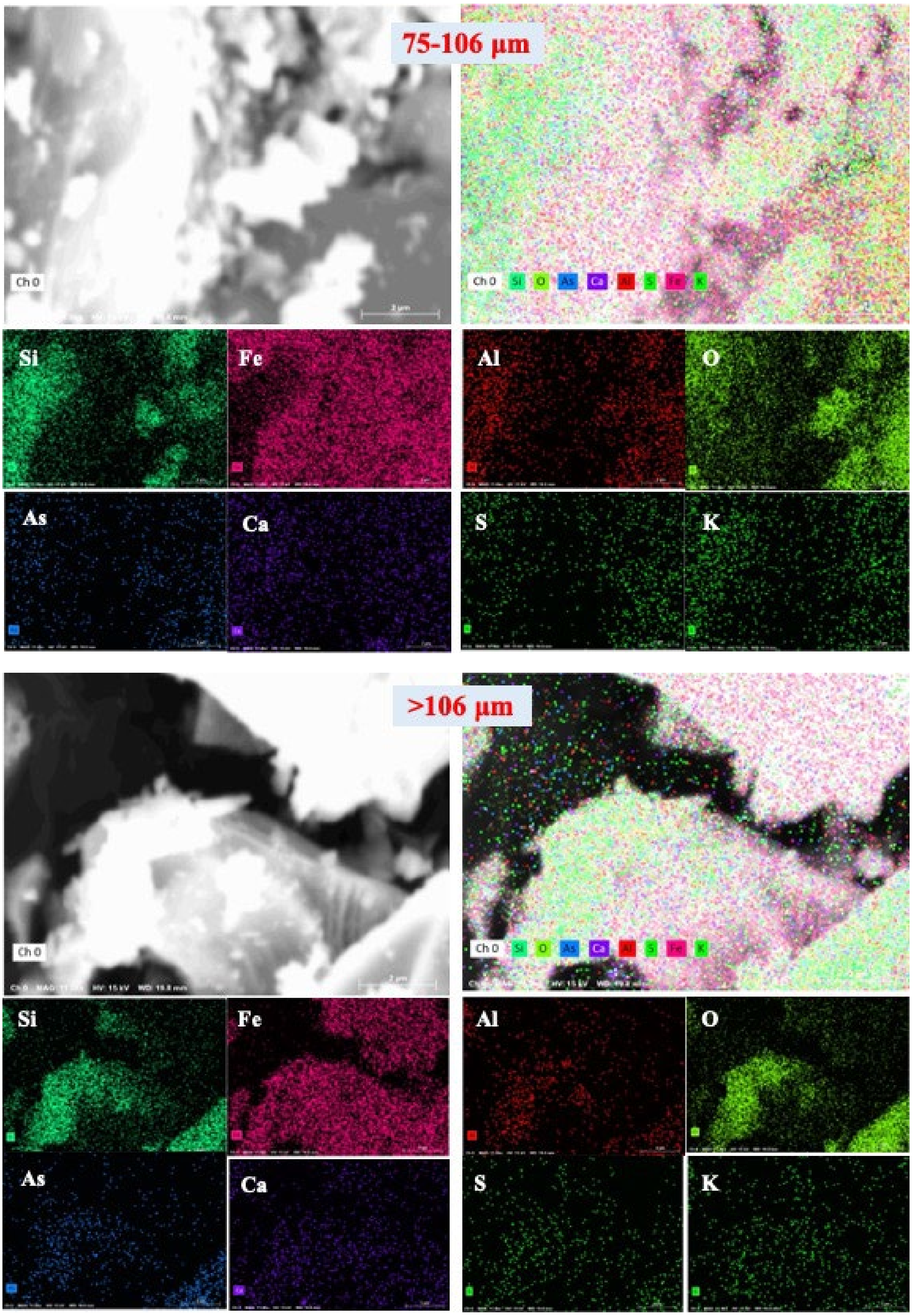

Figure S1. Two-dimensional distribution of elements in different size fractions of slag tailing.
